# Student evaluation of clickers in a dental pathology course

**DOI:** 10.4317/jced.52299

**Published:** 2015-07-01

**Authors:** Carmen Llena, Leopoldo Forner, Roger Cueva

**Affiliations:** 1 MD, DDS, PhD, Department of Stomatology. Universitat de València, C/ Gascó Oliag, 1. 46010 Valencia, Spain; 2 DDS, Department of Stomatology. Universitat de València, C/ Gascó Oliag, 1. 46010 Valencia, Spain

## Abstract

**Background:**

The purpose of this study was to evaluate the degree of satisfaction of students and teachers, and to determine whether the students notice improvements in learning and in the learning environment as a result of the use of clicker.

**Material and Methods:**

Descriptive study. Fifty-one students and 8 teachers participated in the use of clicker technology in 8 preclinical seminars in dental pathology. Students and teachers filled a three-domain questionnaire at the end of the preclinical course. We used the Mann-Whitney U-test to compare the results between the two groups.

**Results:**

The domain “perception and expectation” showed the use of clickers to be simple and convenient for 80% of the students, who expressed interest in extending the practice to other teaching areas. In the domain “active learning”, over 70% of the students found the technique to be dynamic, participative and motivating. In the domain “improved learning”, over 70% considered it useful to know their level of knowledge before the seminar and found the contents of the lesson to be clear. Thirty percent considered the items of the examination to be of a complexity similar to that of the first and second tests. Only in this latter aspect were significant differences found between the teachers and students (*p*=0.001).

**Conclusions:**

Participants described the use of clickers as simple and useful, motivating and participative. Both the students and teachers considered the technique to improve teaching and the learning environment.

** Key words:**Dental education, audience response system, clickers, classroom response system, student´s perception.

## Introduction

The assessment of learning outcomes is the subject of research in teaching and involves the most objective evaluation possible of the competencies acquired by the student in the learning process (1).

Learning represents a genuine change in the knowledge, skills and attitudes of the students. The elements that conform the learning outcomes are difficult to define and even more difficult to evaluate. Consequently, specific objectives must be established in different settings, in relation to motor skills and the cognitive sphere (skills, attitudes, competencies), in the meta-cognitive domain (self-evaluation), and in the administrative area of the learning process (time spent, cost/efficacy, flexibility, etc.). Achieving these objectives results in satisfactory learning and therefore in a positive learning outcome. In this respect, the new technologies can be of help in reaching these objectives in all areas of the process (2).

Active learning requires a change in the educational paradigm, centering on the student and improving the interaction between students and teachers (3). The new technologies facilitates such interaction and, in general, are well accepted by the students and contribute to increase their motivation (4).

Classroom Response System (CRS) is a recently introduced educational tool. This technology allows students to respond and obtain feedback through an immediate response system referred to as the “clicker” method. These new systems are currently available on the market, involve very compatible software, are relatively inexpensive, and are easy to use (5,6).

The simplification afforded by the clicker system is an important asset for use in the educational setting. Two types of system are presently available, respectively based on radiofrequency and infrared communication. The former are more indicated for use in large groups, since they are less vulnerable to interferences (7).

A number of authors suggest that these systems have great potential for improving student learning in the classroom, ensuring more active participation (8,9). They allow evaluation during the class, improve attention, increase interaction, create a dynamic atmosphere, and add an enriching environment to communication (5,10,11).

Some authors report the use of clickers in different educational areas, both in and outside the university setting (12,13), and in dental schools too (14-20).

We introduced the clicker system in the third year of the Dental School of the University of Valencia (Valencia, Spain) for the teaching of dental pathology (DP). The aims of the present study are to describe our experience, evaluate the degree of satisfaction of students and teachers, and to determine whether the students notice improvements in learning and in the learning environment as a result of the use of this new technology.

## Material and Methods

The present study used a questionnaire to explore the opinion of dental students and teachers in the University of Valencia on the use of clicker technology in preclinical seminars in dental pathology (DP). It was approved by the ethics committee of the University of Valencia (H1392830490155).

Dental Pathology (DP) forms part of the subject Dental Pathology, Operative Dentistry and Endodontics. The teaching of DP, in the third year of the Dental School, comprises a series of theoretical classes, preclinical seminars, and clinical practices. The present study was carried out in the context of the preclinical seminars, which number 8 in total and include the presentation of clinical, radiological and histological images referred to DP. These seminars were always programmed for after the theoretical classes. In each seminar the students received a guide detailing the most important aspects addressed in the seminar, together with literature sources for consultation purposes.

The study comprised a total of 51 students (all students enrolled in the subject of Dental Pathology) distributed into three groups of 17 students each, and 8 teachers. A teacher, familiarized with the Classroom Response System (CRS) and trained in the use of the specific clickers employed, imparted each seminar, on different days for each group of students; so, we prepared different groups of questions for each group. A single teacher prepared each of the 8 seminars. All the teachers reviewed and discussed the tests together for each of the seminars in order to ensure uniform presentation and complexity.

The first group of questions in each seminar consisted of 10 multiple choice questions prepared for answering with a radiofrequency clicker system (EduClick, NY, USA). Each of the questions had 5 possible answers, of which only one was correct. The questions could involve text or images and text. The students had 45 seconds to answer each of the questions.

**Figure 1:**
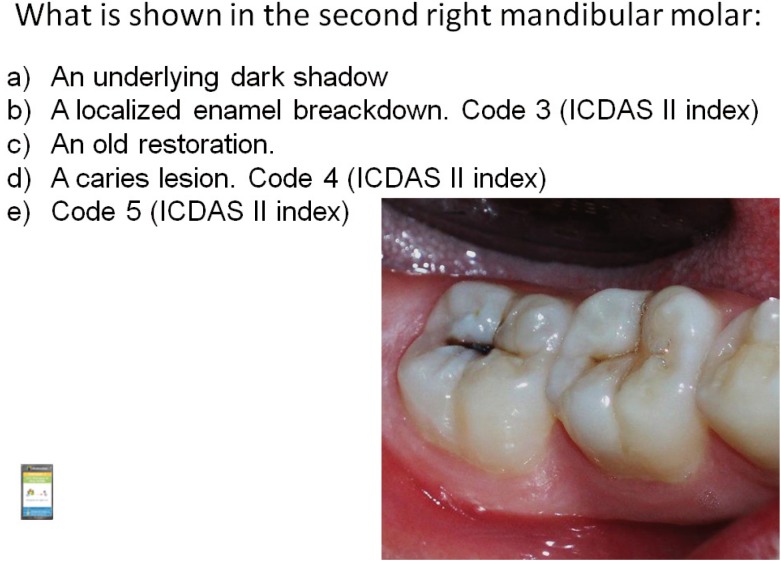
Example of a question test.

Students filled the complete test at the start of the seminar. We performed an immediate checking of the number of right answers for each question, on an anonymous basis. Students and teacher discussed the different answering options for each question in a participative manner, as well as other images, similar to those presented in the test, shown by the teacher. We repeated the same group of questions to be answered with the clicker system, so the students could check the evolution of their group in terms of the number or right answers, on an anonymous basis. We analyzed questions correctly answered by less than 70% of the participants upon repeating the test again. Lastly, we performed an evaluative examination, comprising 15 questions similar to those dealt with during the seminar, using the clicker system. We used this exam to assess learning of the seminar. Figure 1 shows some examples of the questions in the tests.

After completion of the 8 seminars, an 11-item questionnaire evaluated the opinion of the students and teachers regarding the use of the clicker technology and its possible contribution to improved learning. In selecting these 11 items, and following an exhaustive literature review, we established three domains relating to the aspects we wished to explore: 1) “perception and expectation”; 2) “active learning”; and 3) “improved learning”. Then, we evaluated, in a session with all the participating teachers, different items corresponding to each domain for final inclusion in the questionnaire according to the relevance of the contents, simplicity and clarity ( Table 1).

**Table 1:**
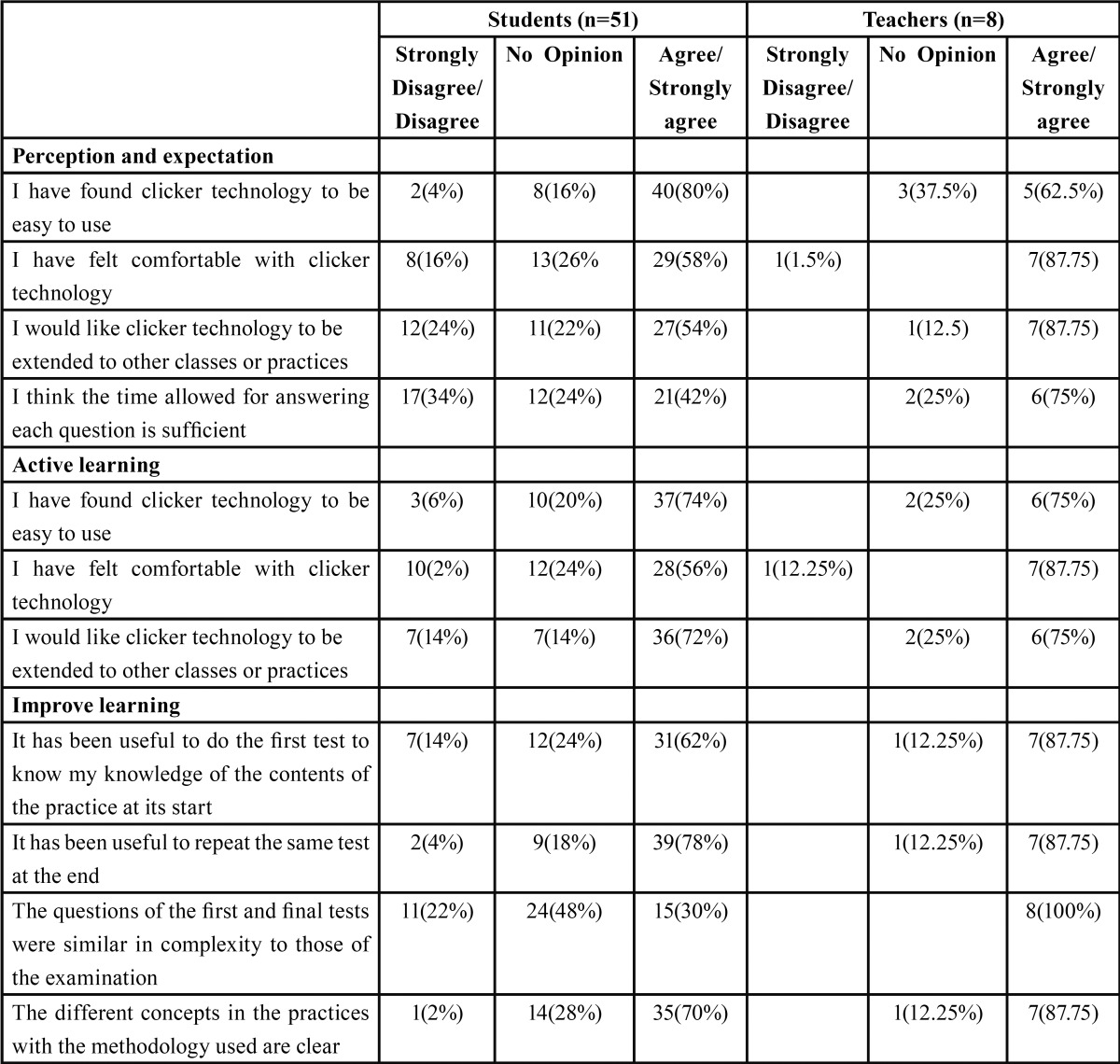
Descriptive analysis of students’ and teacher´s perceptions for each domain tested.

A Likert scale, from 1 (strongly disagree) to 5 (strongly agree), scored each item. A score of 3 represented “no opinion”.

We evaluated internal reliability by Cronbach’s alfa coefficient. We analyzed descriptive data referred to each of the proposed items and we used the Mann-Whitney U-test to compare differences between the students and teachers for each of the items, considering statistical significance for *p* < 0.05.

## Results

The questionnaire response rate among the students was 98.03% (50/51). Eighty-six percent of the participants were females. The mean age of the students was 21.54 ± 4.03 (range 20-37). Reliability, assessed by Cronbach’s alpha coefficient, was 0.887. The reliability of the three domains was 0.773 for “perception and expectation”, 0.860 for “active learning” and 0.787 for “improved learning”.

All of the teachers answered the questionnaire: two of them were full-time professors and the other 6 were associate lecturers. Their mean age was 44 ± 10.02 (range 30-55). Reliability, assessed by Cronbach’s alpha, coefficient was 0.915. The reliability of the three domains was 0.884 for “perception and expectation”, 0.873 for “active learning” and 0.891 for “improved learning”.

 Table 1 shows the descriptive results referred to each of the three domains among the students and teachers.

For students, the domain “perception and expectation” showed the use of clickers to be simple and convenient for 80% of the students, who expressed interest in extending the practice to other teaching areas. In the domain “active learning”, over 70% of the students found the technique to be dynamic, participative and motivating. Lastly, in the domain “improved learning”, over 70% considered it useful to know their level of knowledge before the seminar and found the contents of the lesson to be clear. However, only 30% considered the items of the examination to be of a complexity similar to that of the first and second tests, while 48% had no opinion in this respect.

For teachers, the domain “perception and expectation” showed the use of clickers to be simple and convenient for 62.5% of the teachers, who likewise expressed interest in extending the practice to other teaching areas (87.5% of the teachers). In the domain “active learning”, 75% of the teachers found the technique to be dynamic and participative, and 87.5% considered it to be motivating for learning. Lastly, in the domain “improved learning”, 87.5% considered it useful to perform the first and second tests, and the concepts were considered to have been sufficiently clear. All of the teachers considered the items of the first and second tests to be of a complexity similar to that of the evaluative examination.

The Mann-Whitney U-test only identified significant differences between the teachers and the students in relation to item 10, which assessed similarity of complexity between the first and second tests and the evaluative examination (*p*=0.07).

## Discussion

Learning in dental pathology requires the evaluation of a series of clinical images allowing the student to identify the specific characteristics of the different lesions, with a view to consolidating knowledge and acquiring diagnostic skills. In this respect, seminars involving small groups of students are essential, since they allow the presentation of many images and discussion of the differentiating characteristics of the lesions with other students and with the teachers, before moving on to the clinical practices. The study of dental pathology requires good knowledge of dental anatomy, which is taught in the first year in Dental School. We imparted two introductory seminars to remind the students of dental crown and root morphology.

The introduction of interactive systems in teaching facilitates student attention, and increases curiosity and interaction between the students and teachers (21). It also helps relate new concepts to already acquired knowledge, thereby contributing to reflective learning (22).

We considered reliability, evaluated with Cronbach’s alpha coefficient, to be acceptable for both the global test and for each of the domains, in both the teachers and students, and we considered a coefficient of at least 0.70 to be sufficient for an instrument in its early development stages.

Seventy-four percent of the students found the use of the clickers system to be dynamic, 72% considered it to be a participative procedure, and 60% found the technology to be motivating. These results are consistent with those of other authors (14) in relation to the teaching of periodontics and dental hygiene. Similar findings have also been published in the teaching of pediatric dental care (18).

The capacity to retain information is low in teaching methods in which the student remains passive in class (23). In the case of dental pathology, where the cognitive dimension predominates, the use of instruments designed to improve attention and inter-relate knowledge seems essential in order to improve learning.

Elashvili *et al.* (16) compared two groups, one involving interactive teaching and the other using conventional techniques for learning of the items “principles of dental bonding” and “class IV and V composite resin restorations”. We recorded an increased retention capacity referred to “principles of dental bonding” in the interactive teaching group both at the end of the class and in transferring the knowledge to the practical setting. The students expressed a preference for the interactive system, which they considered to be more effective for understanding the concepts, maintaining attention, and retaining the information. Although our study did not compare two teaching systems in dental pathology, the students were well familiarized with the conventional teaching techniques used in the rest of the academic subjects and produced results similar to those of the mentioned study (16). The use of new technologies should also focus on the affective dimension of the teaching/learning process, which can contribute to increase interest and improve learning (12).

Teacher assessment of the clicker system largely coincided with that of the students. The only exception in this respect corresponded to the item: “The questions of the first and final (the evaluative one) tests were of a complexity similar to that of the examination”, where all of the teachers considered complexity to be similar versus only 30% of the students. On the other hand, 48% of the students claimed to have no opinion in this respect. We founded no studies comparing teacher and student opinion referred to clicker systems, though this probably can be explained by different levels of experience in diagnosis.

The teachers, who had imparted seminars in dental pathology in the past, using conventional methods (i.e., without the clicker system), found the students to be more attentive during the seminars with the new system, more participative, and better able to consolidate knowledge. Indeed, in subsequent seminars that made use of concepts taught in previous seminars, the students showed greater knowledge and did not repeatedly ask the same questions.

The students found clickers useful to perform a first test in the seminar to know their level of knowledge, followed by a repetition of the test at the end. They also considered the concepts of the seminar to be clear with the use of the clicker system.

The use of clicker technology in the teaching of dental pathology is simple and useful, and should be extended to other subjects as well. In view of the results obtained, it would be advisable to extend this study and perform follow-up evaluations over coming academic courses.

## Conclusions

Students and teachers described the use of clickers as dynamic, motivating and participative. The majority of students and teacher were satisfied with the use of this technology and noticed improvements in learning dental pathology as a result of their use.
